# A longitudinal ^1^H-NMR metabolomics analysis of urine from newborns with hypoxic-ischemic encephalopathy undergoing hypothermia therapy. Clinical and medical legal insights

**DOI:** 10.1371/journal.pone.0194267

**Published:** 2018-04-18

**Authors:** Emanuela Locci, Antonio Noto, Melania Puddu, Giulia Pomero, Roberto Demontis, Cristina Dalmazzo, Antonio Delogu, Vassilios Fanos, Ernesto d’Aloja, Paolo Gancia

**Affiliations:** 1 Department of Medical Sciences and Public Health, University of Cagliari, Cagliari, Italy; 2 Department of Surgical Sciences, University of Cagliari, and Neonatal Intensive Care Unit, Puericulture Institute and Neonatal Section, Azienda Ospedaliera Universitaria, Cagliari, Italy; 3 Neonatal Intensive Care, Neonatology, ASO S. Croce e Carle, Cuneo, Italy; Imperial College London, UNITED KINGDOM

## Abstract

Perinatal asphyxia is an event affecting around four million newborns worldwide. The 0.5 to 2 per 1000 of full term asphyxiated newborns suffer from hypoxic-ischemic encephalopathy (HIE), which is a frequent cause of death or severe disability and, as consequence, the most common birth injury claim for obstetrics, gynaecologists, and paediatricians. Perinatal asphyxia results from a compromised gas exchange that leads to hypoxemia, hypercapnia, and metabolic acidosis. In this work, we applied a metabolomics approach to investigate the metabolic profiles of urine samples collected from full term asphyxiated newborns with HIE undergoing therapeutic hypothermia (TH), with the aim of identifying a pattern of metabolites associated with HIE and to follow their modifications over time. Urine samples were collected from 10 HIE newborns at birth, during hypothermia (48 hours), at the end of the therapeutic treatment (72 hours), at 1 month of life, and compared with a matched control population of 16 healthy full term newborns. The metabolic profiles were investigated by ^1^H NMR spectroscopy coupled with multivariate statistical methods such as principal component analysis and orthogonal partial least square discriminant analysis. Multivariate analysis indicated significant differences between the urine samples of HIE and healthy newborns at birth. The altered metabolic patterns, mainly originated from the depletion of cellular energy and homeostasis, seem to constitute a characteristic of perinatal asphyxia. The HIE urine metabolome changes over time reflected either the effects of TH and the physiological growth of the newborns. Of interest, the urine metabolic profiles of the HIE non-surviving babies, characterized by the increased excretion of lactate, resulted significantly different from the rest of HIE population.

## Introduction

Perinatal asphyxia is an event that occurs at a frequency of 1 to 6 per 1000 in countries with high levels of socio-economic development [1]. The hypoxic-ischemic encephalopathy (HIE) has an incidence of 0.5 to 2 per 1000; it is a frequent cause of death and severe disability in survivors. The American Academy of Pediatrics (AAP) and the American College of Obstetricians and Gynecologists (ACOG) stated that HIE is considered as a cause of cerebral palsy (CP) if the following four essential criteria are met: umbilical artery blood metabolic acidosis (pH < 7.0 and base deficit ≥ 12 mmol/L), moderate to severe neonatal encephalopathy, outcome in spastic or dyskinetic tetraparesis, and absence of other causes of CP [2]. Following these criteria, about 14% of children with CP has been exposed to an hypoxic-ischemic brain damage during labour [3].

HIE is most often noted in term newborns. Preterm infants can also suffer from HIE, but the clinical manifestations and pathology are different, involving subcortical grey matter injury in association with white matter damage [4]. Much progress has been made in preventing anoxic event through the centralization of high-risk pregnancies in level III care centres, the accurate monitoring of pregnancies, and the application by trained personnel of the modern techniques of neonatal resuscitation, leading to a more specialized approach to the problem of asphyxia as a whole. In the absence of a causal therapy, the treatment of HIE has been symptomatic for years, aimed at the control of the signs and symptoms secondary to multiorgan anoxic damage and/or failure. Indeed, besides brain damage, muscle, liver, kidney, and heart dysfunction can lead to death even in 50% of cases [5].

Recently, therapeutic hypothermia (TH), a brain cooling technique maintained for 72 hours after the hypoxic-ischemic event, has gained consensus for the treatment of perinatal asphyxia [6]. TH is safe, the incidence of side effects is low, and its implementation is relatively simple from the technical point of view. Brain damage and neuronal death depend on an evolving process that begins during the hypoxic-ischemic insult and it continues, in severe and/or prolonged cases, into the next phase of reperfusion. In the acute phase of the hypoxic-ischemic insult, cellular hypoxia causes a depletion of cellular energy metabolism (primary energy failure) with direct neuronal necrosis. The lack of oxygen prevents oxidative phosphorylation and causes the switch to anaerobic metabolism (glycolysis) with a lower production of adenosine triphosphate (ATP). This energy substrates shortage leads to a rapid consumption of the ATP reserve, accumulation of lactic acid, and inability of the cells to maintain their functions. However, many neurons do not die during the first phase of the hypoxic-ischemic insult, but after reoxygenation of the newborn, during reperfusion of the ischemic brain tissue, which begins after a latency period of at least 6 hours (more specifically, from 6 to 100 hours after the insult). In the reperfusion phase, neuronal death mostly occurs by apoptosis, a mechanism that may last for several days. TH must therefore start within the first 6 hours of life, before the onset of the delayed neuronal damage. In Italy the decision to submit a newborn with HIE to TH requires three consecutive steps to assess his clinical, neurological, and electrophysiological status. These steps have been defined by protocols of randomized controlled trials and, more recently, by the Recommendations of the Italian Society of Neonatology [7]. Systematic reviews and meta-analyses of numerous controlled clinical trials have shown a reduction in mortality and long-term neurodevelopmental disability at 12–24 months of age, with more favourable effects in the less severe forms of HIE [8,9].

From the medical legal point of view, HIE is the most common birth injury claim, based on the allegation that a foreseeable intrapartum asphyxial event is responsible of serious long-term neurologic sequelae [10]. The malpractice claims concerning the neonatal cerebral hypoxia damage are among the ones with the higher economic settlement in the legal scenario (an average indemnity payment of $ 1.030.151 in the USA) [11]. Scientific debate in the courtroom generally relies on the presence/absence of early signs of fetal intrapartum hypoxia-ischemia, which–if adequately evaluated by physicians–could avoid the lengthening of the fetal hypoxia/anoxia and the resulting cerebral damage. Medical malpractice litigation has nowadays reached worldwide a tsunami-like proportion; in the neonatal field, the AAP stated that near one-third of its members has been sued while the ACOG reported an average of three lawsuits per member (2.59), being neurologically impaired infant claims the primary allegation in the obstetric scenario (little more than one out of four of the 1.117 claims) [12]. It is well known to all the expert witness who testified in court that the main conundrum in this context is to objectify the causal relationship between an intrapartum asphyxial event and the neonatal brain damage with a special focus on timing of injury’s onset [13]. The criteria proposed by the Task Force convened by the ACOG and the AAP–and its relevant checklist–represent a helpful tool to reject, but are still unsuitable to assert, the existence of a causal relationship. As clearly stated in the second edition of the report ‘Neonatal encephalopathy and neurologic outcome’ the consensus panel determined that ‘knowledge gaps still preclude a definitive test or set of markers that accurately identifies … an infant in whom neonatal encephalopathy is attributable to an acute intrapartum event’ [14].

Metabolomics is an emerging discipline that allows to identify and quantify the low molecular weight metabolites present in biological fluids and to correlate them with a physiological or a pathological status of the organism [15]. Metabolites represent the molecular endpoint (or phenotype) of the interplay between environment and gene expression and thus metabolomics offers a holistic approach for the early and accurate evaluation of changes in dynamics of biological systems. Recently, metabolomics applied on animal and human models of perinatal asphyxia has raised interest on the research of metabolic pathways related to hypoxia, aiming at identifying metabolic profiles with a potential of outcome predictors [16–30].

In this work, we used a metabolomics approach based on ^1^H NMR spectroscopy combined with multivariate statistical analysis to perform a longitudinal evaluation of the urine metabolome of asphyxiated newborns suffering from HIE and subjected to 72 hours of TH, with the aim of investigating the metabolomics profile at birth and the metabolic modifications occurring over time, from birth up to one month of life. To this purpose, urine samples from 10 newborns, consecutively admitted to the Neonatal Intensive Care Unit, who suffered a perinatal asphyxia and met the HIE diagnostic clinical criteria to be candidate to TH, were collected at different time points: at the admission (within 6 hours from birth), during the TH (at 48 hours), at the end of the TH (at 72 hours), and after one month. Urine samples were also collected at birth from 16 healthy term newborns used as control population.

## Materials and methods

### Study population

This study included a population of 10 full term asphyxiated newborns admitted to the Neonatal Intensive Care Unit of S. Croce e Carle Hospital (Cuneo, Italy), who met the Italian Recommendations to undergo TH [7]. Neonatal characteristics such as gestational age, length and weight at birth, mode of delivery, Apgar scores at 1 min and 5 min were recorded and are summarized in Table 1. HIE was classified according to the Sarnat Grading Scale, based on the infant clinical findings and neurologic signs [31]. All the newborns were treated within the first six hours from birth with a TH regimen over 72 hours; in the same temporal window all the babies were treated with a double antibiotic regimen (ampicillin and gentamicin), while 7 out of 10 babies received phenobarbital.

**Table 1:** Clinical parameters of the study population at birth.

Patient ID	Gender (M/F)	GA (weeks)	BW (grams)	Mode of delivery^a^	Apgar10 min^b^	pH^b^	BE^b^
**U001**	M	38.7	2,530	IVD	0	1	1
**U003**	M	41.1	3,585	ECS	0	1	2
**U004**	M	41.0	3,305	ElVD	2	2	2
**U005**	M	40.0	4,690	OVD-VE	2	2	2
**U006**	M	38.1	3,850	OVD-VE	2	2	2
**U007**	M	39.0	3,910	EVD	0	0	0
**U008**	M	36.0	3,220	EVD	2	2	2
**U010**	F	39.3	3,590	ElVD	2	2	2
**U011**	F	39.8	3,440	OVD-VE	1	2	2
**U012**	F	40.4	3,950	ElVD	2	2	2

**Abbreviations:** GA = Gestational Age; BW = Birth Weight; BE = Base Excess.

^**a**^VD = Vaginal Delivery; IVD = Induced Vaginal Delivery; EVD = Emergency Vaginal Delivery; ElVD = Elective Vaginal Delivery; ECS = Emergency Caesarian Section; OVD-VE = Operative Vaginal Delivery with Vacuum Extractor.

^b^An arbitrary classification scale was used to codify Apgar10min, pH and BE

**Code 0** = Apgar10min ≥ 7; pH ≥ 7,20; BE ≥ -12.

**Code 1** = 5 < Apgar10min < 7; 7 < pH <7,20; -17 < BE < -12.

**Code 2** = Apgar10min ≤ 5; pH < 7; BE ≤ -17.

Urine samples were collected at different time points: at birth (within 6 hours from birth, before starting TH, HIE day 1) and at different times after birth (48 hours–HIE day 2, 72 hours–HIE day 3, and 30 days–HIE day 30). The study protocol was approved by the Independent Ethical Committee of Azienda Ospedaliero-Universitaria of Cagliari (CA-206-18/03/2013), and written informed consent was obtained from both the parents before enrolment in the study.

Clinical data of the patients included: evaluation of brain injury based on pH, base excess, cerebral function monitoring, electroencephalogram, creatine phosphokinase, head ultrasound, cardiac echocardiogram, cardiac troponin, pre and post-TH arterial lactate, kidney function. Head ultrasound scans were performed on day 1, day 2–3, around day 10 and in some cases around day 30 of life, while Magnetic Resonance Imaging (MRI) was performed within the first week and at the end of the first month of life. These time-points were chosen to exclude non hypoxic-ischemic brain injuries (day 1 of life), to assess early patterns of injury (day 2–3 of life), and to define the extent of definitive brain damages (around day 10 and around day 30 of life). Any ventilation, pressor support, or sedation was maintained during the MRI scanning process; additional sedation was avoided [32]. In Tables 1 and 2, the main clinical features of the newborns are reported. An arbitrary classification scale was used for stratifying the qualitative data. As can be seen, 3 out of 10 asphyxiated newborns died within the first week of life (patient ID: 04, 10 and 12). Sixteen healthy full term newborns were also enrolled in the study and their urine samples within 6 hours after birth were used as control population. The HIE and the control populations were matched for gender (7M and 3F in the HIE group; 11M and 5F in the control group), method of delivery (caesarian *vs*. vaginal delivery ratio 1/9), gestational age (39.3 ± 1.5 weeks in the HIE group; 39.6 ± 1.4 weeks in the control group), and birth weight (3607 ± 565 g in the HIE group; 3565 ± 264 g in the control group). Gestational age and birth weight are expressed as mean ± standard deviation. No statistical differences were found between the two groups (unpaired Mann-Whitney U-test was used for inter-groups comparison, and intergroup differences were considered statistically significant at the level of P<0.05). Statistical data analysis was performed by using GraphPad Prism 6.0 (GraphPad Software, La Jolla, CA).

**Table 2:** Clinical parameters of the study population in the first 30 days of life.

Patient ID	CFM^a^	EEG^a^	MRI 1^a^	MRI 2^a^	CPK^a^	Troponin^a^	Pre-TH arterial lactate^a^	Post-TH arterial lactate^a^	Kidney function^a^	Cardiac US^a^	Follow-up
U001	1	1	1	1	2	0	2 (50)	1 (10)	1	0	Mild motor posture delay at 9 months
U003	0	1	0	0	1	na	2 (109)	2 (21)	0	0	Normal
U004	2	2	2	exitus	1	1	2 (112)	2 (18)	2	2	Deceased
U005	1	1	0	0	2	2	2 (130)	1 (13)	0	0	Normal
U006	1	1	1	1	na	na	2 (43)	1 (13)	1	0	Normal
U007	0	0	0	0	0	0	2 (58)	0 (6)	1	1	Normal
U008	0	1	1	0	1	2	2 (66)	1 (14)	1	0	Normal
U010	2	2	2	exitus	2	2	2 (154)	2 (41)	2	2	Deceased
U011	1	1	1	0	2	1	2 (36)	0 (5)	0	0	Normal
U012	1	2	2	exitus	2	2	2 (185)	2 (25)	2	2	Deceased

**Abbreviations:** CFM = Cerebral Function Monitoring; EEG = ElectroEncephaloGraphy; MRI = Magnetic Resonance Imaging; CPK = Creatine Phospho Kinase; US = UltraSound.

^a^An arbitrary classification scale was used to codify the clinical parameters

**Code 0** = No alterations in CFM, EEG, MRI, Kidney function and Cardiac US; CPK ≤ 1000 (U/L); Troponin < 0,20 (ng/mL); Arterial lactate < 7 (meq/L).

**Code 1** = Moderate alterations in CFM (convulsion or depressed), EEG (slow voltage, rhythm period, foc attacks, peak-wave complexes), MRI, Kidney function (temporary AKI) and Cardiac US; 1000 < CPK < 5000 (U/L); 0,20 < Troponin < 0,50 (ng/mL); 7 < Arterial lactate < 15 (meq/L).

**Code 2** = Severe alterations in CFM (burst suppression), EEG (rhythm period with inactive phases), MRI, Kidney function (severe AKI) and Cardiac US; CPK > 5000 (U/L); Troponin > 0,50 (ng/mL); Arterial lactate > 15 (meq/L).

### Samples collection and preparation

Urine sample were collected by a non-invasive method inserting a ball of cotton into the disposable diaper; urine was then aspired with a syringe and transferred to a sterile 2 mL vial. Some urine samples from the asphyxiated group were missing for several reasons (anuria, death, sample insufficient for analysis). After collection, urine samples were added with a 0.1% w/w aqueous solution of NaN_3_ (Sigma-Aldrich, Milan, Italy) in order to prevent bacterial growth, centrifuged at 12000 rpm for 10 min to remove any solid debris, and supernatants were rapidly stored at -80°C until analysis. Before NMR analysis, the urine samples were thawed on ice and centrifuged for 10 min at 12000 rpm at 4°C. 630 μl of the supernatants were mixed with 70 μl of a 1.5 M phosphate buffer solution (pH = 7.4) in D_2_O (99.9%, Cambridge Isotope Laboratories Inc., Andover, MA, USA) containing the internal standard sodium 3-(trimethylsilyl)propionate-2,2,3,3,-*d*_4_ (TSP, 98 atom % D, Sigma-Aldrich, Milan) at a 0.58 mM final concentration, and transferred into a 5mm NMR tube.

### ^1^H NMR experiments

^1^H NMR experiments were carried out on a Varian UNITY INOVA 500 spectrometer (Agilent Technologies, CA, USA) operating at 499.839 MHz for proton. All the spectra were acquired at 300K using a standard 1D-NOESY pulse sequence for water presaturation with a mixing time of 1 ms and a recycle time of 6 sec. Spectra were recorded with a spectral width of 6000Hz, a 90° pulse, and 128 scans. Prior to Fourier transformation the free induction decays (FID) were multiplied by an exponential weighting function equivalent to a line broadening of 0.5 Hz and data were zero-filled to 64K. Chemical shifts were referred to the TSP single resonance at 0.00 ppm. All spectra were phased and baseline corrected using MestReNova software (Version 9.0.0, Mestrelab Research S.L.).

### Data processing and multivariate statistical analysis

The ^1^H NMR spectral regions 1.16–9.00 ppm were reduced into consecutive integrated spectral regions (bins) of 0.02 ppm width using MestReNova. After the removal of water region, drug peaks, and noise a total of 179 bins were obtained. The integrated area within each bin was normalized to a constant sum of 100 for each spectrum in order to minimize the effects of variable concentration among different samples. The final matrix was imported into the SIMCA-P+ program (Version 13.0, Umetrics, Umea, Sweden), and submitted to mean centering and Pareto scaling, before performing multivariate data analysis. Principal Component Analysis (PCA) and Orthogonal Partial Least Square Discriminant Analysis (OPLS-DA) models were applied. PCA, which is the most commonly used unsupervised method, was applied to identify peculiar clusters, anomalies or trends in the samples based on the similarities of their metabolic profiles. OPLS-DA, which is a supervised classification technique, was used to identify variables responsible for the discrimination of predefined classes. OPLS-DA employs linear regression, including the class membership of the samples in the calculation. In OPLS-DA, class separation is maximized in the predictive component (t[1], x-axis) and the orthogonal component (to[1], y-axis) represents the intra-class variability. The number of components for the models was optimized using cross validation. The model quality was evaluated based on R^2^Y (goodness of classification) and Q^2^Y (goodness of prediction) determined through the default leave-1/7th-out cross validation test. The model was tested for over-fitting using y-table permutation testing (n = 400) on the corresponding Partial Least Square Discriminant Analysis (PLS-DA) using the same number of components.

## Results

The clinical parameters of the newborns are reported in Tables 1 and 2, together with main laboratory and instrumental data. Despite the early onset of TH regimen and a standard pharmacological assistance, 3 HIE newborns died within the first 7 days after birth for causes attributable to HIE and comorbidity conditions (patient ID = 04, 10 and 12). For the surviving babies, the application of the Bayley-III scale of infant and toddler development demonstrated the absence of any neurodevelopmental abnormality at the end of the first month of life.

All the collected urine samples were analysed by ^1^H NMR spectroscopy. The obtained NMR spectra showed several resonances originating from the different functional groups of the low-molecular weight water-soluble metabolites detectable in the samples. Assignment of the resonances was based on literature data [33]. Major resonances originated from free amino acids, organic acids, small organic compounds, osmolytes, and sugars.

In order to investigate similarities or differences among samples on the basis of their metabolomics profiles, the binned NMR spectral data were submitted to multivariate data analysis. At first, an unsupervised PCA was applied to all the collected samples to obtain an overview of their distribution and to detect possible sample clustering and/or outliers.

**Fig 1:**
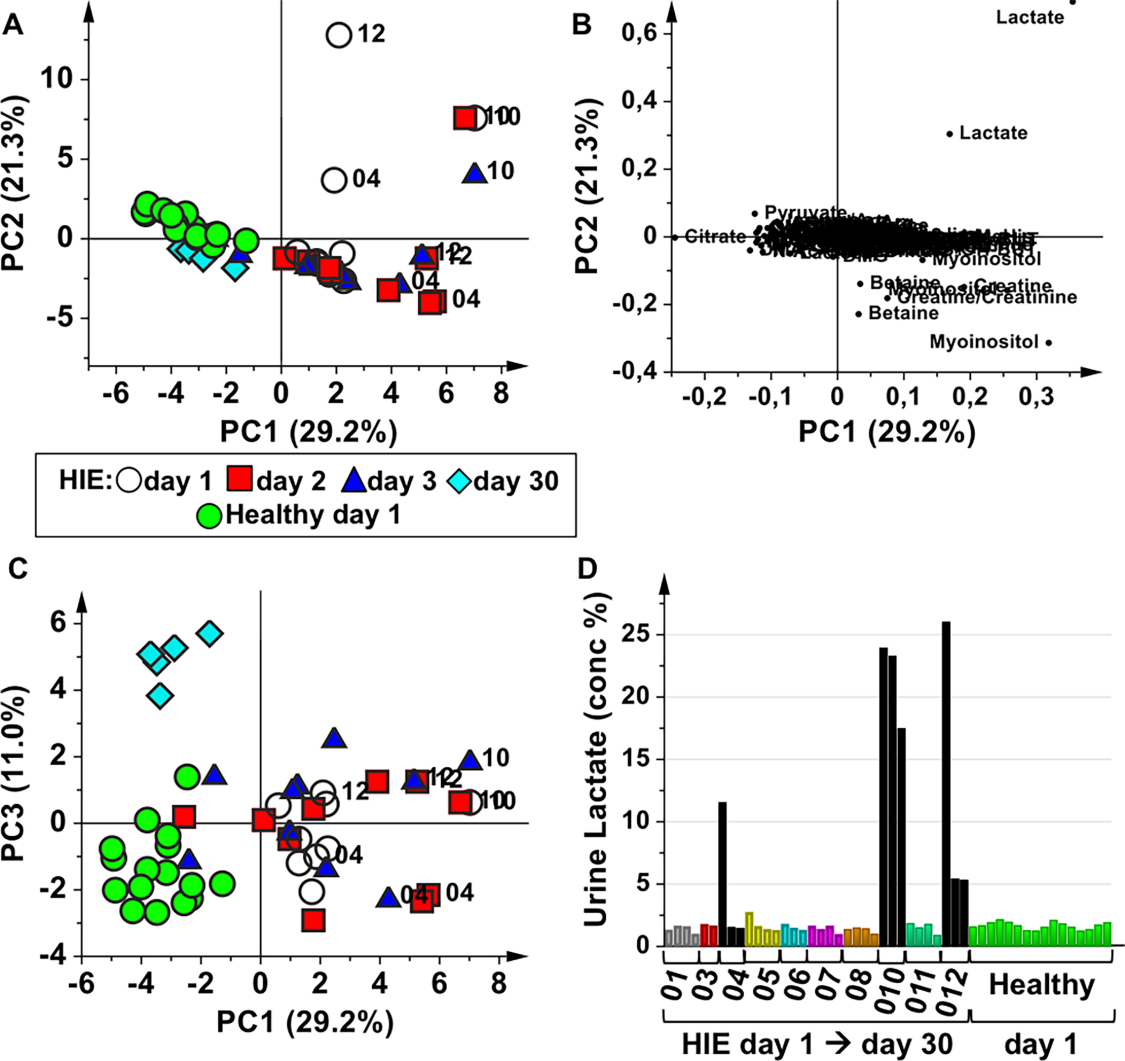
Overview of the collected urine samples from HIE newborns and healthy controls. PCA model of urine samples collected from HIE newborns at birth (HIE day 1, open circles) and at different time points after birth: HIE day 2 (48 hours during TH, red squares), HIE day 3 (end of the TH, blue triangles), HIE day 30 (light blue diamonds), and from healthy control newborns at birth (Healthy day 1, green circles). **(A)** score plot of PC1 *vs* PC2; **(B)** loading plot of of PC1 *vs* PC2; **(C)** score plot of PC1 *vs* PC3; **(D)** urine lactate relative concentration, obtained from the integrated area of the spectral region 1.32–1.36 ppm normalized to the total integrated area. For HIE babies urine lactate relative concentration is reported for all the sampled time-points (from day 1 to day 30, except for non-surviving newborns), while only the concentration at birth is reported for the control newborns.

Fig 1 represents the PCA model of urine samples collected from HIE newborns at different time points: at birth (HIE day 1), at 48 hours during the TH treatment (HIE day 2), at 72 hours that corresponds to the end of TH (HIE day 3) and at 30 days after birth (HIE day 30), together with the urine samples collected from the healthy control newborns at birth (Healthy day 1). The first three PC’s explained the 61.5% of the total variance. In the score plot of PC1 *vs* PC2 (Fig 1A) the urine samples belonging to HIE newborns are distributed along a time-related trajectory. In particular, from day 1 to day 30 the HIE urine samples move in the multivariate space toward the region where the healthy control samples lie, indicating modifications of the HIE metabolomics profiles. Urine samples of asphyxiated newborns that did not survive (patient ID: 04, 10 and 12) are quite well distinguished from the remaining samples. The analysis of the PCA loading plot (Fig 1B) indicates that the urine metabolome of non-surviving HIE newborns is characterized by higher amounts of lactate with respect to the samples of surviving HIE newborns. Interestingly, the score plot of PC1 *vs* PC3 (Fig 1C) shows that HIE day 30 samples and healthy control samples are not overlapped, as it could appear in Fig 1A, but instead they form two separate clusters, indicating that the corresponding metabolic profiles are different. The urine samples belonging to one non-surviving baby (ID = 10 –samples at 1, 2, and 3 days) lie in the same multivariate space, indicating that no major metabolic modifications occurred during time despite the TH treatment, whereas the samples of the other two non-surviving babies (ID = 04 and 12) seem to undergo metabolic modifications during the second and third day after the birth. This behaviour is mainly related to the urinary lactate concentration of these samples. Indeed, as shown in Fig 1D, the urine lactate relative concentration of the 3 time-points of patient ID = 10 remained high and almost constant until the death of the baby, whilst in the samples of patients ID = 04 and 12 urinary lactate decreased over time. It is worth to note that the surviving HIE newborns showed at birth similar urinary lactate relative concentration with respect to healthy control babies.

**Fig 2:**
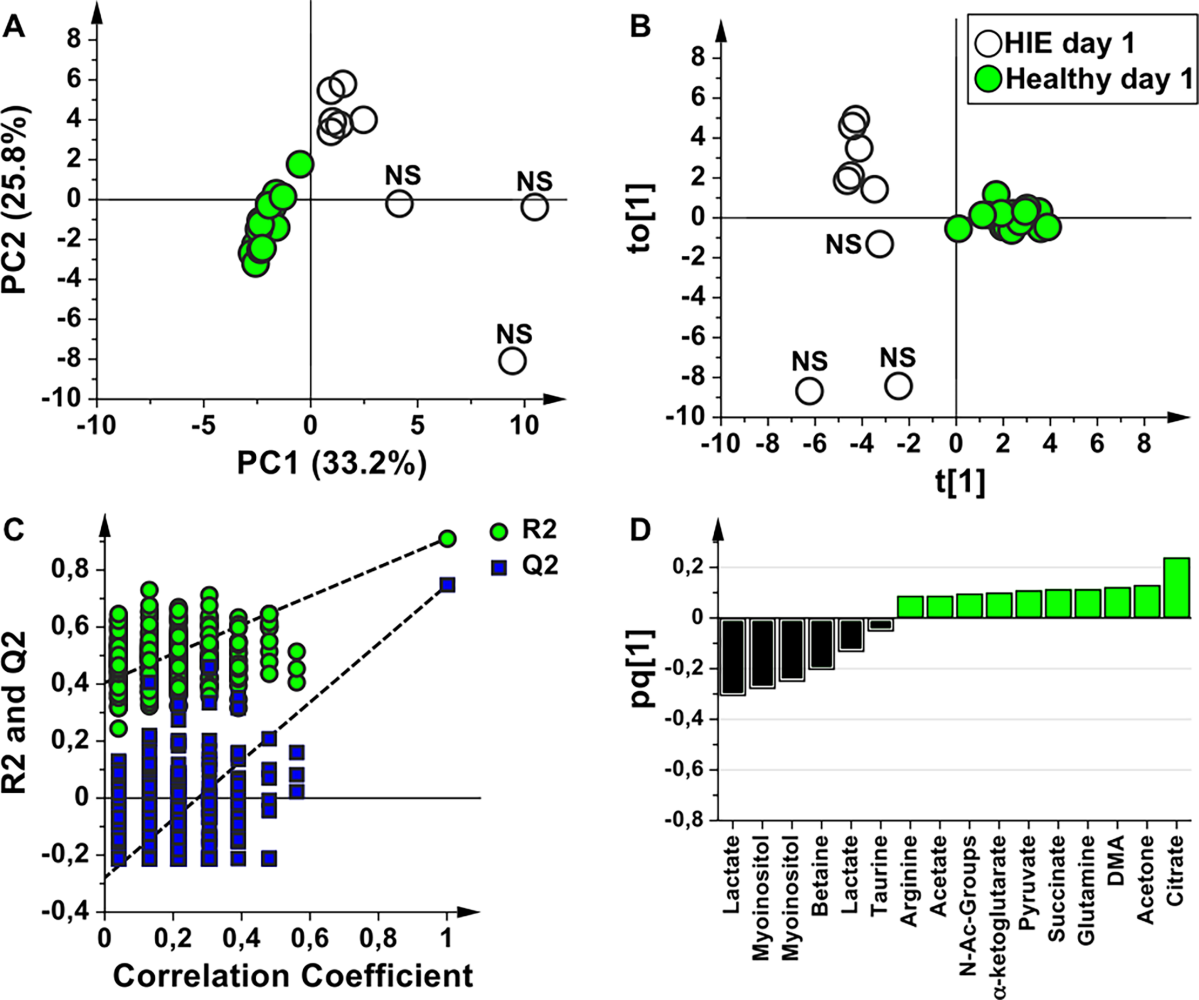
Comparative analysis of urine samples of HIE newborns and healthy controls at birth. Score scatter plots of (**A**) PCA and (**B**) OPLS-DA models of urine samples collected from HIE newborns (HIE day 1, empty circles) and from healthy newborns (Healthy day 1, green circles). The label NS indicates the non-surviving babies. (**C**) Permutation test of the corresponding PLS-DA model (n = 400 random permutations). (**D**) OPLS-DA loading column plot of discriminant variables.

In order to identify a urinary metabolomics profile characteristic of asphyxiated newborns, the HIE samples at birth were compared with those of the healthy controls. The score scatter plots of PCA and OPLS-DA models are shown in Fig 2. HIE newborns and healthy control samples are well separated in the PCA (Fig 2A, explained variance by the first three components 68.3%), indicating that are characterized by a different metabolic profile. The OPLS-DA model (Fig 2B) showed A = 1+1 components, R^2^Y = 0.91, and Q^2^ = 0.75. In order to validate the OPLS-DA model, a permutation test was performed on the corresponding PLS-DA model (Fig 2C); the test showed that all the Q^2^ and R^2^ values of the permutated models were lower than the original values (n = 400, intercepts for R^2^ = 0.41 and Q^2^ = -0.28). HIE and control samples are well separated along the predictive component (t[1], x-axis) further indicating a distinct urine metabolome. A deeper insight in Fig 2B indicates that control samples are very well clustered, whereas HIE samples are stratified along the orthogonal component (to[1], y-axis) that models the within group (intra-class) variability. This orthogonal distribution is related to the severity of HIE or to the comorbidities developed by newborns as a consequence of the asphyxial period, the two subgroups corresponding to the surviving and the non-surviving babies, respectively. The metabolites that are altered in the HIE with respect to the control samples are indicated in the OPLS-DA loading plot (Fig 2D). The HIE group is characterized by the relative increase of lactate, myo-inositol, and betaine, while TCA cycle intermediates (citrate, α-ketoglutarate, succinate), acetone, dimethylamine (DMA), glutamine, pyruvate, N-Acetyl groups, arginine, and acetate are relatively lower with respect to controls. If the 3 non-surviving HIE newborns are excluded from the analysis, the HIE surviving newborns remain separated from the healthy group. The metabolite alterations are the same previously identified, except for lactate, which does not appear anymore as a discriminating metabolite.

**Fig 3:**
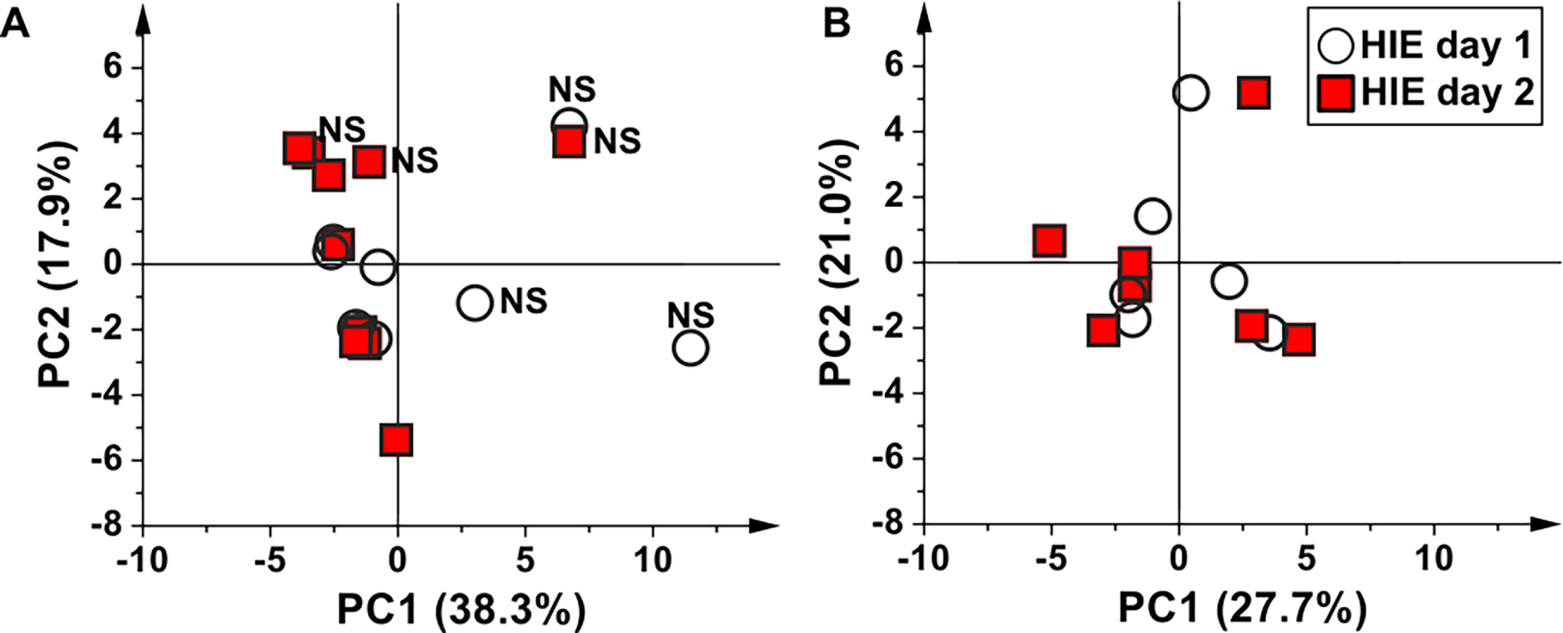
The effect of 48 hours of TH on the urine metabolome of HIE newborns. Score scatter plots of (A) PCA model of urine samples collected from HIE babies at birth (HIE day 1, empty circles) and during the TH treatment at 48 hours (HIE day 2, red squares). The label NS indicates the non-surviving babies, and (**B**) corresponding PCA model after removal of the samples belonging to non-surviving newborns.

In order to investigate the modifications of the urine metabolome in HIE newborns during TH, at the end of TH and at 30 days of life, several two-groups models were generated where the samples collected at each time point were individually compared with the samples collected at birth (Fig 3). Fig 3A shows the PCA score plot of HIE urine samples collected at birth (HIE day 1) and at 48 hours after birth (HIE day 2). The first two components explained the 56.2% of the total variance. The samples belonging to the non-surviving HIE newborns lie once again apart from the others. If we exclude these latter from the analysis (Fig 3B, explained variance by the first two components 48.7%), HIE day 1 and day 2 samples are randomly distributed, suggesting that during the first 48 hours, despite the TH treatment no significant metabolic modifications occurred. This assumption is supported by the fact that no validated supervised model could be built.

**Fig 4:**
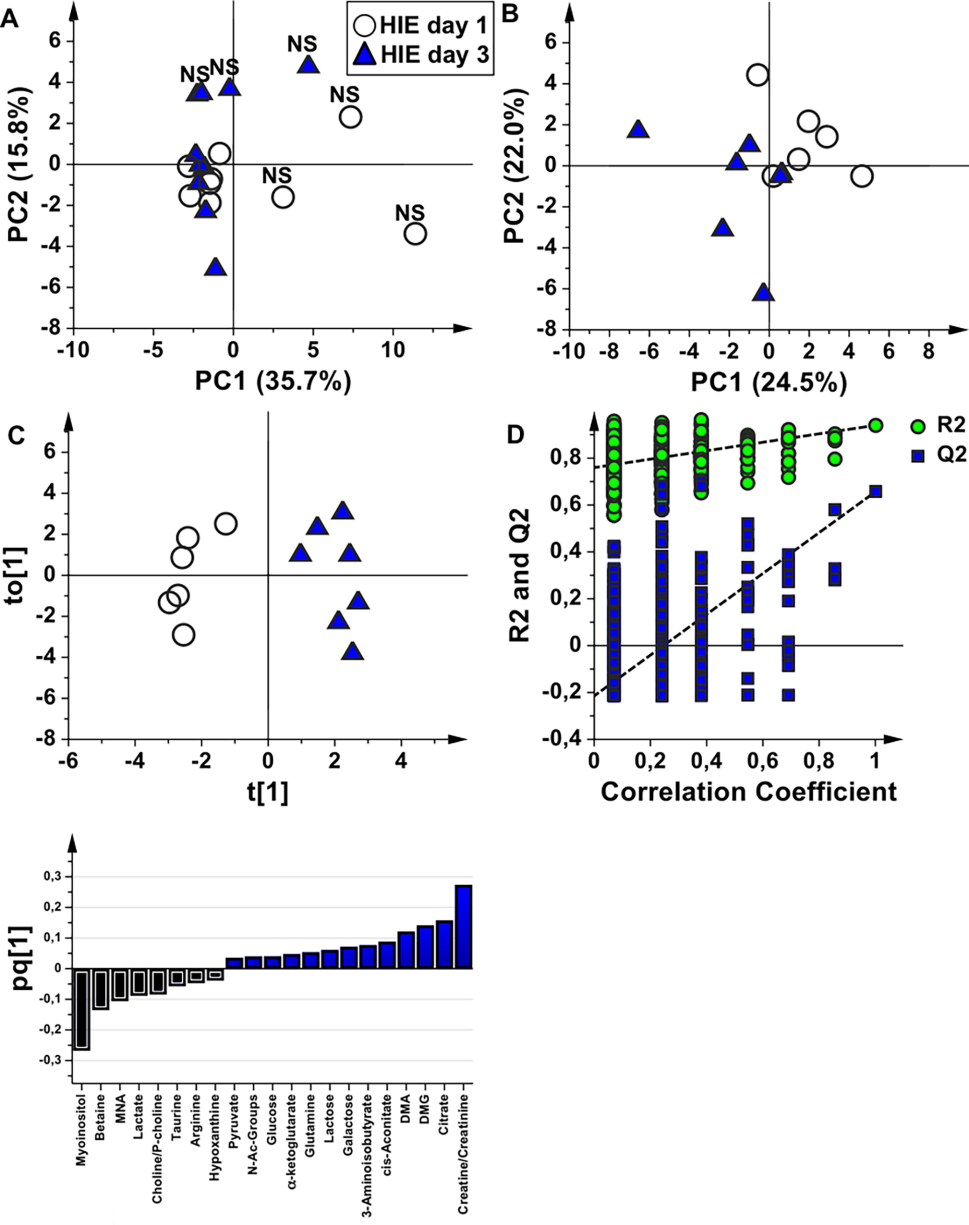
The effect of 72 hours of TH on the urine metabolome of HIE newborns. (**A**) Score scatter plot of the PCA model of urine samples collected from HIE newborns (HIE day 1, empty circles) and at the end of TH treatment (HIE day 3, blue triangles). The label NS indicates the non-surviving newborns. (**B**) Score scatter plot of the PCA model after removal of the samples belonging to non-surviving newborns (**C**) Score scatter plot of the OPLS-DA model of the samples belonging to surviving newborns. (**D**) Permutation test of the corresponding PLS-DA model (n = 400 random permutations). (**E**) OPLS-DA loading column plot of discriminant variables.

HIE samples at birth (HIE day 1) were then compared with those collected at the end of the TH treatment (HIE day 3). In the PCA score plot (Fig 4A, explained variance by the first two components 51.5%) the samples belonging to the non-surviving babies are, once again, clearly distinguishable from the others. However, if the samples of the non-surviving newborns are eliminated from the analysis, HIE samples at the end of the TH treatment are separated from the corresponding samples at birth in the PCA score plot (Fig 4B, explained variance by the first two components 46.5%). This is different from what observed after 48 hours of TH, where the samples remained randomly distributed, and indicates modifications of the metabolic profile after complete TH. In order to identify these modifications, only the samples belonging to the surviving newborns were used to generate the OPLS-DA model (Fig 4C, A = 1+1 components; R^2^Y = 0.94, Q^2^ = 0.66; Fig 4D, permutation test n = 400, intercepts for R^2^ = 0.76 and Q^2^ = -0.22). At the end of the TH treatment, samples were characterized by higher amounts of creatine, citrate, N,N-dimethylglycine (DMG), DMA, cis-aconitate, 3-aminoisobutyrate, galactose, lactose, glutamine, α-ketoglutarate, glucose, N-Acetyl groups and pyruvate and lower amounts of myo-inositol, betaine, 1-methyl-nicotinamide (MNA), lactate, choline/phosphocholine, taurine, arginine, and hypoxanthine with respect to HIE samples at birth (Fig 4E).

**Fig 5:**
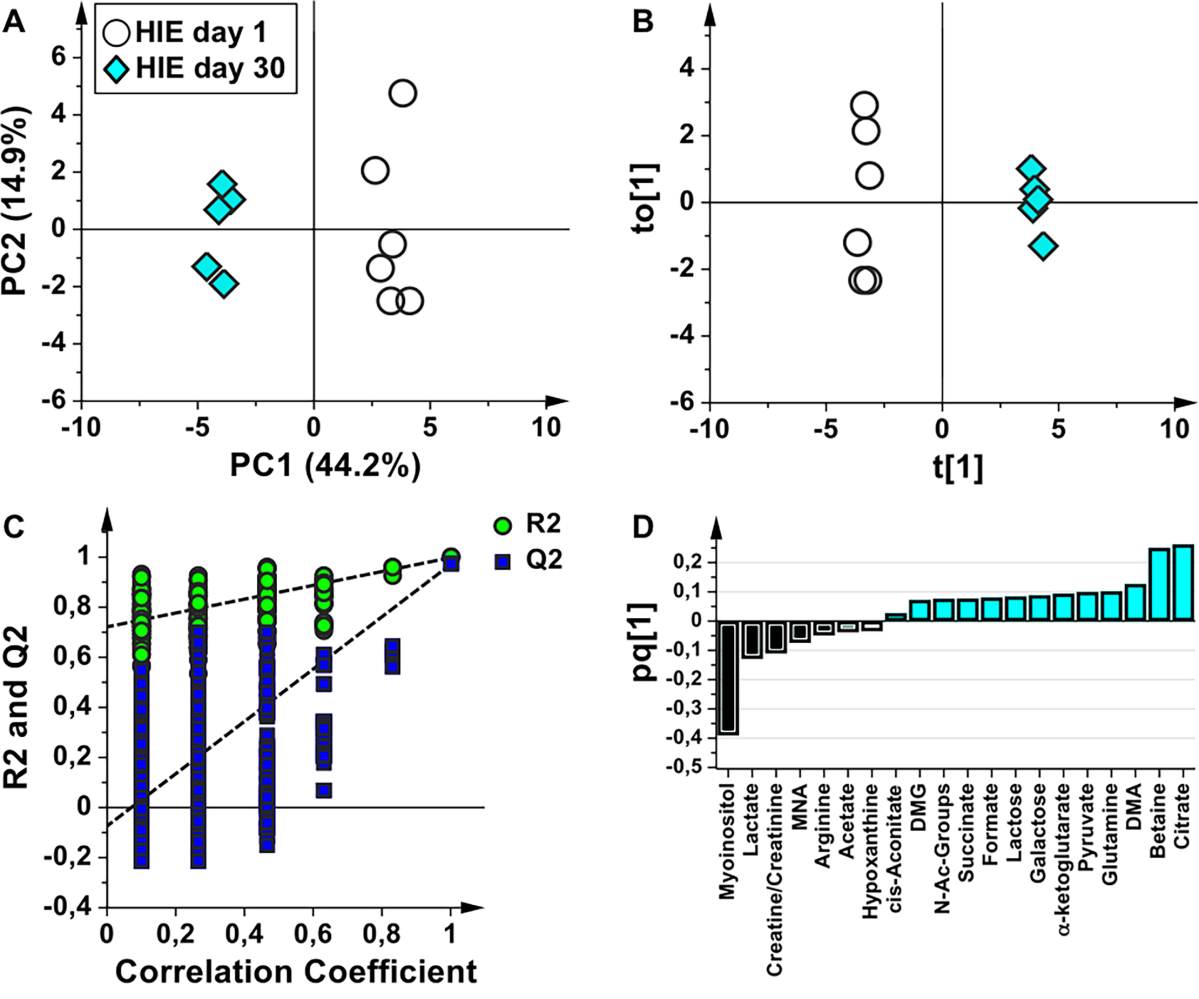
Comparative analysis of urine samples of HIE newborns at birth and at 30 days of life. Score scatter plots of (**A**) PCA and (**B**) OPLS-DA models of urine samples collected from HIE babies at birth (HIE day 1, empty circles) and at 30 days of life (HIE day 30, light blue diamonds). (**C**) Permutation test of the corresponding PLS-DA model (n = 400 random permutations). (**D**) OPLS-DA loading column plot of discriminant variables.

At 30 days of life a consistent shift of the metabolic phenotype of the survived HIE newborns compared with the samples at birth was found. The PCA and OPLS-DA models are shown in Fig 5. Samples are well separated in the PCA score plot (Fig 5A, explained variance by the first two components 59.1%). The OPLS-DA model (Fig 5B, A = 1+1 components; R^2^Y = 0.98, Q^2^ = 0.97) further indicates a clear separation between the two groups and resulted validated by the permutation test (Fig 5C, permutation test n = 400, intercepts for R^2^ = 0.72 and Q^2^ = -0.07). The corresponding loading plot (Fig 5D) indicates that at 30 days there was a higher excretion of TCA cycle intermediates, betaine, DMA, glutamine, pyruvate, galactose, lactose, formate, N-Acetyl groups, and DMG, while the metabolic profile at birth remained characterized by the same metabolites previously identified in the comparison between HIE day 1 and day 3 samples with the only exception of betaine (which characterizes the HIE 30 days profile), and of creatinine and acetate (more expressed in HIE 1 day samples).

## Discussion

### Clinical aspects

The key question, addressed by the HIE newborns parents to the neonatologist in the early hours of the infant life, obviously deals with the prognosis both in terms of survival and of neurodevelopmental outcome. HIE long-term outcome may include cognitive delays, motor disabilities, and a broad spectrum of health problems. To date, there is no unique prognostic marker, but it is the sum of the instrumental and clinical data that may help in predicting the future of the child. Early clinical outcome predictors include the stage of encephalopathy and the persistence of other neurological abnormalities after a week or more from birth. Long-term outcome criteria include standardized test (Bayley-III) that may assess child cognitive and language skills and fine and gross motor development even at the first month of life. In the mean time, amplitude-integrated EEG is a reliable outcome predictor in cooled infants 48 hours after the asphyxial event. MRI, starting from the first week of life, is the more useful tool to assess the severity of brain injury and to provide prognostic information in cooled HIE newborns. MRI morphological patterns are recognized and classified into 5 categories, which can be extremely reliable outcome predictors [34, 35]. Today, trustworthy brain-specific serum or urine biomarkers are not available. S100B protein and neuron-specific enolase (NSE) have been proposed and they seem to be good outcome predictors only before TH [36].

In this scenario, the use of metabolomics seems of importance since it may offer the possibility to identify peculiar patterns of biomarkers allowing to increase the sensitivity and specificity and to characterize the metabolic fingerprint of the disease. The design of the experiment included only term infants, with HIE defined using both Sarnat Grading Scale and EEG, studied at different time points after birth. The overall PCA model shown in Fig 1A clearly suggests a dynamic change over time in the urine metabolome of HIE newborns treated with TH going from birth to one month of life. The healthy control group lies apart in the multivariate space. Within the HIE group, non-surviving newborns are clearly separated from the surviving ones due to a different metabolome, mainly characterised by an increased urinary lactate. During the asphyxial event, the activation of the anaerobic glycolysis pathway leads to an increased production of lactate, the accumulation of which induces acidosis, one of the parameters used to determine the need of TH. In non-surviving HIE newborns, the accumulation of urinary lactate at birth over the semi-quantitative threshold limit indicated in Fig 1C seems to be, in our restricted sample, an irreversible condition. Lactate is an alternative source of energy for neurons, which can switch from oxidative to non-oxidative metabolism due to reduced availability of oxygen. Some studies also indicated lactate as a predictor of worst outcome, but its prognostic role, although promising, did not reach a full consensus yet [37–39]. In particular, the time of normalization of plasma lactate in cooled HIE babies showed a 23.1 h median value (range 2.9–73.5 h) in babies with good outcome and a 39.1 h median value (range 8.1–134.1 h) in the ones who survived with a poor outcome [39]. Moreover, a recent MR spectroscopy imaging study performed in a neonatal piglets hypoxic-ischemic brain injury model showed that, after the hypoxic-ischemic insult, the basal ganglia lactate content increase immediately, it reaches its maximum value at 2–6 hours, and then gradually decreases to the level of the control group [40]. In our sample, although all the babies were diagnosed with HIE Sarnat II-III and fulfil the criteria adopted from the Italian Recommendations for TH, urinary lactate relative concentration in HIE surviving newborns showed a similar trend over time whenever compared with healthy newborns, suggesting the existence of a wide variability either in the causes of HIE or in individual responses to hypoxic-ischemic insult. Although an increase in urinary lactate was reported in gentamicin treated animals, its nephrotoxic effect induces complex metabolomics modifications that were not observed in our HIE population [41].

The dynamic changes over time in the urine metabolome of HIE newborns reasonably reflect either the effects of TH (especially in the first 72 hours) and the physiological growth of the newborns (in the first month). Using as starting point the urine metabolome at birth, major metabolic changes in the HIE population are summarized in Table 3. The data indicate the existence of a trend in the HIE urine metabolome. As increased excretion of TCA cycle intermediates (citrate, α-ketoglutarate, cis-aconitate, and succinate), pyruvate, DMA, DMG, lactose and galactose is observed in HIE newborns during the first month of life, while lactate, myo-inositol, hypoxanthine, choline/phosphocholine, arginine, and MNA decreased over time.

**Table 3:** Metabolomics data. Major metabolic changes in the HIE population over time.

HIE day 1 compared to Healthy day 1		HIE day 3 compared to HIE day 1		HIE day 30 compared to HIE day 1	
Increased	Decreased	Increased	Decreased	Increased	Decreased
Lactate	Citrate	Creatine/Creatinine	Myo-inositol	Citrate	Myo-inositol
Myo-inositol	Acetone	Citrate	Betaine	Betaine	Lactate
Betaine	DMA	DMG	MNA	DMA	Creatine/Creatinine
Taurine	Glutamine	DMA	Lactate	Glutamine	MNA
	Succinate	cis-Aconitate	Choline/P-Choline	Pyruvate	Arginine
	Pyruvate	3-aminoisobutyrate	Taurine	α-Ketoglutarate	Acetate
	α-Ketoglutarate	Galactose	Arginine	Galactose	Hypoxanthine
	N-Ac groups	Lactose	Hypoxanthine	Lactose	
	Acetate	Glutamine		Formate	
	Arginine	α -Ketoglutarate		Succinate	
		Glucose		N-Ac-Groups	
		N-Ac groups		DMG	
		Pyruvate		cis-Aconitate	

DMA = dimethylamine; MNA = 1-Methyl-Nicotinammide; DMG = N,N-dimethylglycine

Many of the metabolites here identified are related to the cellular energy metabolism. Citrate, α-ketoglutarate, cis-aconitate, and succinate, which are intermediates of the TCA, are involved either in the mechanism of cellular respiration or may have a role as marker of cellular damage [42]. The increase of these metabolites over time could be also related to a progressive re-activation of aerobic pathways after the hypoxic-ischemic event. This is also suggested by the progressive reduction of urinary lactate over time. In anaerobic conditions, lactate is produced via pyruvate, an intermediate of the glycolysis, while in an aerobic context, it supplies energy to the cell through the TCA. Hypoxanthine, a metabolite of purine cycle, is also accumulated during hypoxia, due to gradual ATP depletion. Hypoxanthine may act as substrate for the formation of radical oxygen species resulting in local and systemic oxidative stress through lipid peroxidation. The presence of ketone bodies, such as acetone and acetate, is expected during the neonatal period in which lipid metabolism is more active. Ketone bodies are produced in the liver from the oxidation of fatty acid, but are needed for brain metabolism since no other non-glucose source can be used [43]. The presence of DMA and DMG, which indicate the activation of the methylamine metabolism, seems to be related to their conversion in taurine or pyruvate. Taurine is an osmolyte involved in the maintenance of intracellular homeostasis and in the balance of neurotransmitters. The presence of other osmolytes, such as myo-inositol, betaine and choline, is also of importance, as their extracellular accumulation at birth in HIE may be due to cellular injury and death due to hypoxia. The urinary decrease of these latter over time in HIE newborns can be therefore explained by the progressive recovery of injured tissues and organs. Choline, beyond being involved in the physiologic control of the osmotic pressure, is a precursor of phosphatidylcholine which is needed for signalling and transport across membranes and is an intermediate by-product of membrane component disruption, being related to membrane damage due to cell acidification and ROS production [44]. The presence of the glutamine-glutamate metabolic cycle is believed to be vital for preventing neuronal excitotoxicity. Neuroprotective effects of astrocytes are not only dependent on glutamate uptake but also on glutamine synthetase activity. For the latter, the capacity for storage and release of glutamine in astrocytes is critical [45]. MNA, a metabolite of nicotinamide, was found to have anti-inflammatory properties [46]. Finally, the presence of lactose and galactose is certainly due to feeding of the newborns.

Summarizing these results, some issues may be addressed. First, in our sample a polymorphic clinical scenario (see Tables 1 and 2), although all the babies fulfil the clinical conditions for a HIE diagnosis and meet the criteria for a TH regimen, is represented. From a metabolomics point of view, it is possible to discriminate from the urine metabolome at birth the 3 newborns that will eventually die from the surviving ones, showing an average neuropsychological development in the first month of life. The metabolomics profile is dominated by the surge of urinary lactate at birth. When the semi-quantitative measure of lactate is compared among the 10 HIE newborns and the 16 healthy controls, an intriguing result may be drawn. Only the non-surviving HIE newborns presented a ‘pathological’ lactate semi-quantitative level, while the corresponding values in the 7 surviving HIE babies was similar to the healthy controls. Thus, the pathogenesis of the HIE may be different between the two groups of HIE babies, depending either by the severity of the hypoxic insult or by the biological cause of the asphyxia (it may be hypothesized a ‘less’ acute insult in the babies with a ‘relative’ lower concentration of urinary lactate or a not obstetrical cause of it). If this assumption will be confirmed in a wider cohort, a metabolomics approach will be very helpful in discriminating the ‘peripartum’ causes of asphyxial encephalopathy (which seems to be in the relevant literature a residual portion of the whole phenomenon) from all the others ‘non-medical’ ones. Secondly, even if the lactate contribution to the HIE profile is elided (especially for the HIE no-surviving group), a different profile between the HIE and healthy newborns at birth underlying the fact that the metabolomics response of HIE newborns to asphyxia/hypoxia has its own clear signature.

Finally, in the HIE surviving group some consideration has to be devoted, to the comparison between the metabolomics profiles at 48 and 72 hours after birth. In Fig 3A and 3B it is clear that at 48 hours, no significant metabolite modifications occurred–although a standard pharmacological assistance and a TH is administered to all the newborns, and no discriminant analysis could be performed. A clear-cut difference between the profiles was instead appreciated at the end of the treatment, suggesting that the 72 hours period for the hypothermia, chosen on an empirical basis, may have a biological explanation, being this temporal window needed for the treatment to begin its biological effect. Once again, this research had not this issue as its principal endpoint and an *ad hoc* experiment should be drawn to address this intriguing clinical aspect. Moreover, it is possible to hypothesize the development in the next future of a diagnostic kit that could also help for the inclusion of off label patients, such as preterm infants, or for the extension of TH application beyond the 6 hours as suggested in the guidelines.

### Medical liability aspects

The preliminary results of this study, although not useful to draw a general inference on the pathogenesis of HIE, seem to discriminate among several causes of neonatal encephalopathy suggesting a relevant role of lactate in the abrupt onset of severe asphyxia/hypoxia while all the other causes of HIE or degree of intrapartum hypoxia may be responsible of a metabolomics profile closer to the ‘normal’ adaptive response to delivery, although still recognizable.

The advent of TH, and its proven beneficial role in the treatment of certain newborns and in the improvement of neonatal neurological outcomes, has deeply modified the neonatologist attitude towards neonates born with moderate to severe HIE. This new approach and the clinical guidelines pertaining to its use have the collateral effect to widen the liability horizon to the neonatologists. The clinical selection of babies who may benefit from TH, the local availability of the equipment, and the strict timing for neonatal submission to the treatment, are some of the areas where the failure to fulfil the clinical criteria may be considered reasons behind new allegations of medical liability. In the longitudinal analysis here presented, the behaviour of neonatal urinary metabolome during the TH regimen was analysed, showing a clear changing trend in babies who eventually benefit from the treatment. Even from this point of view, the identification of a personalized metabolomics profile at birth–and during the TH regimen–should be useful for the medico-legal evaluation of individual response of HIE babies. The profile–and its time-related modification—may be used either to support or to reject the hypothesis that a more well-timed treatment should, or should not, be able to change the natural clinical history of that baby or to confirm that the TH has gained its clinical goal, being the neurological sequelae in no either way amendable. Further and wider studies are needed to validate these assumptions but a metabolomics approach seems to be *a priori* advantageous to unravel this complex puzzle and to confirm/negate the causative role of the culprit event (namely a medical misconduct whenever an intrapartum asphyxia has been identified).

The results and the conclusions of our study should be cautiously interpreted due to the following limitations. First, the size of our HIE sample was too small to draw general inferences on the pathophysiological events underlying this complex clinical scenario. We performed the analysis on such a limited number of little patients due to the need to have all the babies treated in the same way by a selected physicians group in order to reduce all the clinical and the pharmacological biases. For this reason, all the biological samples were collected in a single Neonatal Intensive Care Unit. A wider sampling is needed to confirm the results here reported and a multicentre study is currently underway. Second, we are aware about the incomplete representation of the control group, which should include together with the healthy samples also biological specimens from TH untreated HIE Sarnat II-III babies. It is obvious either that the urinary metabolomics profile of those babies should be different by the ones here depicted and may be very helpful to draw the metabolic pathways activated in a hypoxic-ischemic milieu, and that it is ethically unfair to deprive a group of newborns of a mandatory treatment. Maybe it could be possible to collect urine from HIE Sarnat II-III babies who do not fulfil the clinical criteria to undergo TH, such as, for example, all the ones who are transferred from the birth place to the Hospital in a time period longer than the therapeutic window provided for by National and International guidelines, even though these cases are nowadays residual. Finally, the lack of a clinical and metabolomics follow-up of the HIE newborns at 1 year of life or more prevents from drawing any possible inference concerning the clinical effect of the hypoxic event on their neurological and behavioural development.

### Conclusions

Perinatal asphyxia is a complex and multifactorial disease. Rather than focusing on the research of single biomarkers, the use of a holistic multivariate approach such as the one proposed in the current study could help in the identification of a HIE specific metabolomics profile.

The advantage of the urine analysis by ^1^H NMR metabolomics is related to the rapid non-invasive and non destructive assessment of the final products of the metabolism: this could be useful for monitoring early cellular injuries and death during a hypoxic-anoxic insult and for the development of short- and long-term negative outcomes, in order to apply early preventive measure to improve the quality of life of newborns with HIE. Finally, a significant better knowledge of the individualized metabolomics may help to unravel the issue of neurological newborns damage occurring during delivery.

## Supporting information

S1 Table ^1^H NMR data matrix. ^1^H NMR binned spectral data. HIE, HIE newborns; Healthy, healthy newborns; S, surviving HIE newborns; NS, non-surviving HIE newborns.
(XLSX)
